# Cancer Precision-Prevention trial of Metformin in adults with Li Fraumeni syndrome (MILI) undergoing yearly MRI surveillance: a randomised controlled trial protocol

**DOI:** 10.1186/s13063-024-07929-w

**Published:** 2024-02-03

**Authors:** Miriam Dixon-Zegeye, Rachel Shaw, Linda Collins, Kendra Perez-Smith, Alexander Ooms, Maggie Qiao, Pan Pantziarka, Louise Izatt, Marc Tischkowitz, Rachel E. Harrison, Angela George, Emma R. Woodward, Simon Lord, Lara Hawkes, D. Gareth Evans, James Franklin, Helen Hanson, Sarah P. Blagden

**Affiliations:** 1 Department of Oncology, University of Oxford, Old Road Campus Research Building, Oxford, OX3 7DQ UK; 2 Oncology Clinical Trials Office, University of Oxford, Old Road Campus Research Building, Oxford, UK; 3 Trial Support Unit, Department of Oncology, University of Oxford, Old Road Campus Research Building, Oxford, UK; 4 Centre for Statistics in Medicine and Oxford Clinical Trials Research Unit (OCTRU), Nuffield Department of Orthopaedics, Rheumatology and Musculoskeletal Sciences, Headington, Oxford, UK; 5 George Pantziarka TP53 Trust, 7 Surbiton Cres, Kingston upon Thames, UK; 6 Guy’s and St Thomas’ NHS Foundation Trust, Great Maze Pond, London, UK; 7 Department of Medical Genetics, National Institute for Health Research Cambridge Biomedical Research Centre, Cambridge, UK; 8 Department of Clinical Genetics, Nottingham University Hospitals NHS Trust, Hucknall Rd, Nottingham, UK; 9 Royal Marsden Hospital, Fulham Road, London, UK; 10 Division of Evolution, Infection and Genomics, School of Biological Sciences, Faculty of Biology, Medicine and Health, The University of Manchester, Manchester, UK; 11 Manchester Centre for Genomic Medicine, Manchester University NHS Foundation Trust, Manchester, UK; 12 Oxford Centre for Genomic Medicine, ACE building, Nuffield Orthopaedic Centre, Windmill Road, Headington, Oxford, UK; 13 Institute of Medical Imaging and Visualisation, Bournemouth University, St Pauls Lane, Bournemouth, UK; 14 Peninsula Clinical Genetics Service, Royal Devon University Healthcare NHS Foundation Trust, Exeter, UK; 15 Faculty of Health and Life Sciences, University of Exeter, Heavitree Road, Exeter, UK

**Keywords:** Metformin, Li-Fraumeni syndrome, LFS, Cancer, p53, *TP53*, Precision-Prevention, Chemoprevention

## Abstract

**Background:**

Li-Fraumeni syndrome (LFS) is a rare autosomal dominant disease caused by inherited or de novo germline pathogenic variants in *TP53*. Individuals with LFS have a 70–100% lifetime risk of developing cancer. The current standard of care involves annual surveillance with whole-body and brain MRI (WB-MRI) and clinical review; however, there are no chemoprevention agents licensed for individuals with LFS. Preclinical studies in LFS murine models show that the anti-diabetic drug metformin is chemopreventive and, in a pilot intervention trial, short-term use of metformin was well-tolerated in adults with LFS. However, metformin’s mechanism of anticancer activity in this context is unclear.

**Methods:**

Metformin in adults with Li-Fraumeni syndrome (MILI) is a Precision-Prevention phase II open-labelled unblinded randomised clinical trial in which 224 adults aged ≥ 16 years with LFS are randomised 1:1 to oral metformin (up to 2 mg daily) plus annual MRI surveillance or annual MRI surveillance alone for up to 5 years. The primary endpoint is to compare cumulative cancer-free survival up to 5 years (60 months) from randomisation between the intervention (metformin) and control (no metformin) arms. Secondary endpoints include a comparison of cumulative tumour-free survival at 5 years, overall survival at 5 years and clinical characteristics of emerging cancers between trial arms. Safety, toxicity and acceptability of metformin; impact of metformin on quality of life; and impact of baseline lifestyle risk factors on cancer incidence will be assessed. Exploratory end-points will evaluate the mechanism of action of metformin as a cancer preventative, identify biomarkers of response or carcinogenesis and assess WB-MRI performance as a diagnostic tool for detecting cancers in participants with LFS by assessing yield and diagnostic accuracy of WB-MRI.

**Discussion:**

Alongside a parallel MILI study being conducted by collaborators at the National Cancer Institute (NCI), MILI is the first prevention trial to be conducted in this high-risk group. The MILI study provides a unique opportunity to evaluate the efficacy of metformin as a chemopreventive alongside exploring its mechanism of anticancer action and the biological process of mutated P53-driven tumourigenesis.

**Trial registration:**

ISRCTN16699730. Registered on 28 November 2022. URL: https://www.isrctn.com/ EudraCT/CTIS number 2022-000165-41.

**Supplementary Information:**

The online version contains supplementary material available at 10.1186/s13063-024-07929-w.

## Background

Li-Fraumeni syndrome (LFS), also referred to as heritable *TP53*-related cancer (hTP53rc) syndrome, is a rare autosomal dominant cancer predisposition syndrome caused by inherited or de novo germline likely pathogenic or pathogenic variants in *TP53*, henceforth termed *TP53* GPV (germline pathogenic variant). Males and females with classical LFS have around a 70% and 100% lifetime risk of cancer respectively, with around 50% having their first cancer diagnosis before the age of 46 and 31 years. Typical LFS “core” malignancies include bone and soft-tissue sarcomas and very early onset-breast, brain and adrenocortical cancers but a wide phenotypic spectrum of cancers have been reported in LFS families. In the UK, there are estimated to be over 600 people with a known genetic diagnosis of LFS, and with the increasing use of genetic sequencing in diagnostic practice, this number is estimated to rise [1]. In view of the high penetrance for cancers, published guidelines recommend that “heterozygotes” or carriers of *TP53* GPV to undergo yearly cancer surveillance from birth comprised of whole body (WB) + brain (B) magnetic resonance imaging (MRI) alongside breast MRI (for women over 20 years who have not undergone risk-reducing mastectomy), annual clinical review and skin examination [2–4]. Studies demonstrate evidence of improved long-term survival with early tumour detection via surveillance programmes [5, 6]. There are no medications licensed to prevent the onset of cancer in LFS which is a high unmet medical need. Here we present the MILI trial, in which the cancer preventive activity of the antidiabetic drug metformin will be assessed in patients with LFS.

### *TP53* and cancer

*TP53* is described as the “guardian of the genome” for its role in protecting cells exposed to reactive oxygen species (ROS) accumulation, DNA damage and/or oxidative stress. It achieves this by binding DNA, RNA and proteins involved in DNA repair, cell proliferation, senescence, apoptosis, mitophagy, autophagy, metabolism and angiogenesis [7]. Collectively P53 modulates expression or activation of proteins in these pathways to buffer stress in order to ensure that cells survive or, if stress is extreme, undergo programmed cell death (also known as apoptosis).

LFS or hTP53rc syndrome occurs due to an inherited or de novo GPV in *TP53.* Most typical variants are missense variants located within exons 4–9 of the *TP53* gene, which encode its DNA binding domain (DBD). The reason that loss of DNA binding leads to the emergence of cancer is incompletely understood. In the majority of cases, it is thought that a second “hit”, or somatic (acquired) mutation to the remaining wild-type *TP53* allele, termed loss of heterozygosity (LOH), precedes cancer [8]. Alternatively, the mutant p53 protein (mutp53) represses normal p53 protein produced by the wild-type allele through a dominant-negative effect. Some pathogenic variants have also been shown to encode a more stable form of p53 protein with less affinity for its canonical target genes but greater affinity for others such as components of the trimeric 5′ adenosine monophosphate-activated protein kinase (AMPK) [9]. This is termed “gain of function” (GOF) and confers mutp53 with oncogenic capabilities [10]. Individuals with LFS are known to have higher basal levels of oxidative stress compared to unaffected family members and this background may also contribute to increased cancer risk through ROS-induced DNA damage [11].

In 2004, a *Trp53*^*515A*^ knock-in “LFS mouse” was made, carrying a genetic alteration equivalent to a missense pathogenic variant at the R175 hotspot within the DBD [12]. As well as forming spontaneous tumours, mice that were homozygous or heterozygous for this *TP53* GPV showed increased oxidative metabolism synonymous with heightened mitochondrial activity. This finding was replicated in myoblasts obtained from the skeletal muscle of LFS patients after exercise [13]. It is proposed that, without normal p53 protein to balance metabolic outputs, mutated *TP53* de-represses mitochondrial oxidative phosphorylation (OXPHOS) which drives the heightened production of ROS causing DNA damage and mutagenesis. Cells from LFS mice also had enhanced lipolysis and fatty acid synthesis presumed due to the repression of AMPK [14]. When LFS mice were crossed with mice bearing a heterozygous or homozygous pathogenic variant in the mitochondrial gene DNA polymerase gamma, rates of metabolism were lowered and cancer-free survival was increased by 40% and 79% respectively [12]. As the anti-diabetic agent metformin is known to inhibit mitochondrial OXPHOS, mice were given metformin from 4 weeks of age and a reduction in oxidative metabolism markers and delayed time to forming cancer by 27% were observed [12]. As well as its mitochondrial role, *TP53* also negatively regulates PI3K-AKT-mTOR signalling at numerous points by suppressing IGF1R transcription, enhancing transcription of the tumour suppressor protein PTEN and preventing AKT activation [15]. When mutated, as shown in in vivo studies using m*tp53* mice, AKT activity is increased and tumours develop [16]. Further work has shown increased transformation and invasiveness in cells with both PI3K/AKT pathway activation and loss of *TP53* [17, 18]. Hence, loss of *TP53* may release the brake on upstream mitogenic input from insulin and IGF1.

It is likely that other mechanisms may also contribute to cancer formation in LFS. The *tp53*^*515A*^ mouse model, although bearing a typical *TP53* pathogenic variant, forms a different spectrum of cancers (such as sarcomas and lymphomas) than are observed in humans with LFS. Monozygotic twins with LFS do not have similar cancer incidence indicating environmental (such as diet) and immune factors that contribute to cancer risk [14].

### Metformin

Metformin (1,1-dimethylbiguanide) is a synthetic derivative of a natural compound found in French lilac. It is an oral glucose-lowering agent that has been used to treat more than 120 million people with type 2 diabetes since it was first licensed in the 1950s.

Despite its metabolic benefits, metformin can cause gastrointestinal side effects in up to 30% of patients, including diarrhoea, nausea, vomiting, abdominal bloating and anorexia. These symptoms usually occur at the onset of therapy, rarely persist and can be mitigated by careful dose-escalation and, if necessary, de-escalation [19]. Because of its repression of aerobic mitochondrial respiration, pyruvate generated from enhanced glycolysis is converted to lactic acid, and hence, metformin overdose or failure to excrete metformin (90% is renally cleared) alongside difficulties in compensating for raised lactate can cause a fall in body pH and lactic acidosis. This is a rare but serious side effect of metformin with an estimated incidence of 6 cases per 100,000 patient-years [20]. For this reason, contraindications for metformin use include chronic kidney disease, liver disease and heart failure as well as the use of renally cleared intravenous contrast agents within 48 h of dosing. It is standard practice to assess the renal function of any patient starting metformin and to carefully dose-escalate to reach the standard dose of 2g/day [21]. Pharmacodynamic studies have previously shown that the maximal efficacy of metformin occurs at 2 g/day (its recommended dose) as, above this level, drug absorption decreases and the incidence of gastro-intestinal toxicity increases [22].

### Metformin as a cancer preventive in sporadic cancers

**Fig. 1:**
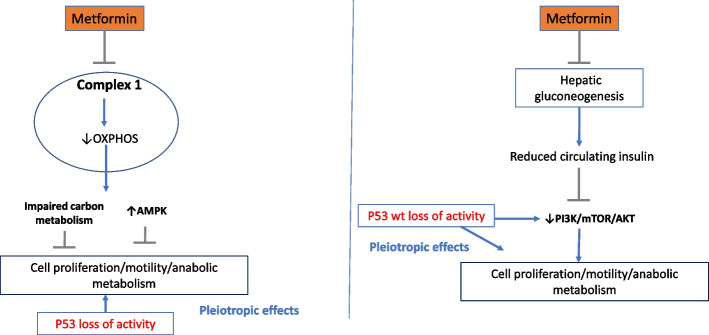
Hypotheses for metformin’s cancer prevention properties. Left-hand panel: Here, metformin directly inhibits Complex 1 of the electron transport chain. The function of Complex 1 is to accept electrons from NADH before donating them to Coenzyme Q for further shuttling down the electron transport chain (ETC). Along with other members of the ETC, the energy released by electron transfer facilitates the pumping of protons across the inner mitochondrial membrane to create an electrochemical gradient that allows ATP synthase to catalyse the conversion of ADP to ATP. Hence, metformin reduces oxidative phosphorylation (OXPHOS) disrupting cellular energy homeostasis and leading to activation of AMPK kinase. AMPK, a key regulator of energy balance, inhibits several anabolic pathways critical for cell proliferation. Additionally, interference with the ETC and tricarboxylic acid (TCA) cycle disrupts carbon metabolism and the supply of macromolecules required for cell proliferation [15]. Right hand panel: Metformin inhibits Complex 1 in hepatocytes activating AMPK and suppressing hepatic gluconeogenesis. It indirectly lowers circulating glucose and insulin levels, downregulating PI3K/AKT/mTOR signalling and suppressing cell proliferation, motility and anabolic metabolism

Preclinical studies have confirmed the potential of metformin as a cancer-preventative agent [12] and randomised clinical trials have now shown that this drug can prevent the development of precancerous lesions [23]. To date, results from trials testing the clinical efficacy of metformin in established cancers have been disappointing [24]. Two main hypotheses have emerged to explain its possible antitumorigenic effect: (i) that metformin directly inhibits mitochondrial function in malignant or premalignant cells or (ii) that it acts indirectly by reducing hepatic gluconeogenesis and hence circulating glucose, insulin and IGF1 levels [25, 26] (see Fig. 1). Population prevention studies do not allow these mechanistic questions to be resolved, as they tend to recruit genetically diverse patients with multiple cancer risk factors and take many years to deliver their primary endpoint of cancer-free survival.

### Pilot trial: short-term administration of metformin to adults with LFS

Following the preclinical findings in LFS mice showing metformin delayed the emergence of cancer, a pilot trial was then conducted in humans with LFS [27]. Twenty-six participants (20 females and 6 males) were enrolled and consented to take metformin for 14 weeks; metformin was given at a starting dose of 500 mg once daily and dose-escalated to 2 g/day in 500 mg increments every 2 weeks. It was generally well-tolerated with only low-grade (grade 1 or 2) side effects such as diarrhoea (affecting 50%), nausea (46%), dyspepsia (19%) and headache (30%). The majority reported complete resolution of these side effects by week 14. No episodes of lactic acidosis were observed. To assess the biological impact of metformin, blood samples were obtained for measurement of serum IGF1, insulin and IGFBP3. Exhaled CO_2_ after ingestion of ^13^C-labelled methionine was measured in participants as a readout of hepatic mitochondrial function. Even though participants were non-diabetic, metformin was shown to lower circulating IGF1 and IGFBP3 levels, increase markers of fatty acid beta-oxidation and reduce levels of exhaled ^13^C-methionine at weeks 8 and 14 compared with baseline. This confirmed metformin is well tolerated and effective at reducing OXPHOS in LFS but did not explore its impact on PI3K/AKT or AMPK levels. The size and short duration of the trial did not allow correlations with subsequent cancer incidence.

The purpose of MILI is to conduct the follow-on trial to this pilot study, to assess the impact of metformin on cancer incidence in adults with LFS and also to interrogate its mechanisms of action.

### International LFS consortium

As well as *TP53* GPV in LFS, somatic mutations to *TP53* occur in up to 50% of cancers, either as an early event driving tumorigenesis (in ovarian cancer for example) or as a late event in established cancers. Germ-line and somatic pathogenic variants tend to occur at similar hotspot sites within the *TP53* gene—predominantly within its DBD. The acquisition of a *TP53* mutation within an existing cancer almost invariably signifies the emergence of treatment resistance, increased tumour proliferation and adverse clinical outcome. It is conceivable that similar molecular processes follow sporadic as well as germ-line *TP53* pathogenic variants. However, the limited opportunity to temporally track these changes in cancer or high-cancer-risk patients has hindered our understanding of *TP53*-driven tumorigenesis. To address this, we intend to collect yearly blood samples from MILI participants to extract PBMCs, plasma and cell-free (cf)DNA to conduct parallel biomarker studies.

Although the recommended screening for all LFS patients in the UK is yearly MRI surveillance [3], it is likely that different *TP53* GPVs may be associated with different cancer risk and/or distinct cancers. For example, a specific *TP53* GPV c.1010G>A p.R337H is enriched in Southern Brazil and is carried by an estimated 1/375 individuals. R337H is located in the C-terminal oligomerisation domain of *TP53*, outside the DBD. R337H was first identified in children with adrenocortical carcinoma, but families rarely fulfilled classical LFS criteria [28]. It is now recognised that individuals with this variant do have an increased risk of LFS cancers, such as breast cancer, but most typically at an older age [29]. More recently, other variants, such as *TP53* c.455C>T p.(Pro152Leu), have been described to be associated with lower penetrance than is typically recognised in LFS [30]. Hence, better genotype-phenotype correlations and improved knowledge of the cancer penetrance associated with specific GPVs could better stratify LFS patients for surveillance.

Due to the rarity of *TP53* GPV, the UK prevalence of LFS is too low to conduct these association studies. Hence, an international LFS consortium has been established with investigators from the National Cancer Institute (NCI, USA), Hannover (Germany) and SickKids (Canada), to conduct parallel MILI studies in the USA and Germany so that data can be pooled to address cancer penetrance. Trial centres in each nation are seeking independent funding to conduct MILI in adults and/or children using a locally adapted core protocol. Each trial will be sufficiently powered to address the primary objective as a standalone trial. Results from all studies will be pooled in an individual participant data (IPD) meta-analysis to definitively determine the benefit of metformin in preventing cancer in this high-risk participant group and address secondary and translational endpoints.

The UK LFS organisation, the George Pantziarka TP53 Trust, has also been involved with the design of MILI at a very early stage of development. In addition to outreach within the LFS community, it has provided feedback and data to support the trial and is continuing a high level of PPI activity.

### Precision-prevention trials

MILI is a Precision-Prevention (PP) trial. PP trials test targeted interventions (such as metformin) in high cancer-risk patients where specific efficacy has been observed. PP trials have three main objectives in patients: (1) whether a targeted intervention is an effective chemo-preventive, (2) the intervention’s mechanism of chemo-preventive action and (3) the molecular process of tumorigenesis in the high-cancer-risk group. To determine whether an intervention (metformin) is effective in the MILI study, we will be comparing clinical outcomes of cancer-free, tumour-free (including non-cancerous lesions such as precancers) and overall survival at 5 years. To address the mechanism of action of metformin and explore tumorigenesis, questions 2 and 3 require these participants in PP studies to undergo serial sampling of blood.

## Methods

The SCRIPT reporting guidelines have been followed for the reporting of the MILI protocol [31].

### Participants, interventions and outcomes

#### Design

The MILI Precision-Prevention trial is an open randomised, non-placebo-controlled phase II trial of metformin 2 g daily versus no metformin in adults with LFS undergoing annual MRI-based cancer surveillance.

#### Setting

The MILI trial is conducted through the UK Cancer Genetics network and integrated into the standard of care surveillance pathways for adults with LFS so that participation in MILI does not affect routine care for those in either trial arm. Recruiting sites will be centres chosen from the UK Cancer Genetics network. This is comprised of 24 UK centres. Patients will be identified by their local genetics centres which will either be recruiting or referring sites.

#### Participants

Eligible participants are aged >16 years old with a germline likely pathogenic class IV or pathogenic class V *TP53* variant (by CanVIG-UK criteria) [32].

Exclusion criteria include:

1. Currently taking metformin
2. Metformin intake for more than 3 months in total, within the 2 years antecedent to the date of trial enrolment
3. Completion of cancer systemic therapy within the 6 months antecedent to the date of trial enrolment
4. Current type 1 or 2 diabetes mellitus
5. Presence of active ongoing cancer (detected previously or at baseline scanning)
6. Current pregnancy or lactation
7. Gastro-intestinal condition (such as short-bowel syndrome) that could affect the absorption of metformin
8. Concurrent medical condition (other than LFS) that could result in life expectancy of <5 years
9. History of the following cardiac conditions:
  - Congestive cardiac failure of > Grade II severity according to the New York Heart Association Functional Classification (defined as symptomatic at less than ordinary levels of activity).
  - Ischaemic cardiac event including myocardial infarction within 3 months prior to date of enrolment.
  - Uncontrolled cardiac disease, including unstable angina pectoris and uncontrolled hypertension (i.e. sustained systolic BP > 160 mmHg or diastolic BP > 90 mm Hg)

10. Evidence of significant renal impairment eGFR < 50 ml/min/1.73 m^2^
11. Liver cirrhosis and/or alkaline phosphatase, aspartate transaminase or alanine transaminase >2.5 × upper limit of normal (ULN)
12. Elevated risk of lactic acidosis such as current chronic alcoholism, congenital lactic acidosis and concurrent intake of carbonic anhydrase inhibitor (e.g. acetazolamide)
13. Known allergy to metformin
14. Does not fulfil MRI Safety Screening criteria (e.g. implanted cardiac pacemaker, post-surgical metal hardware—plates, etc.) and/or unable to undergo baseline scan.

Note that ongoing use of aspirin, fish oils or other health supplements are not exclusions.

#### Intervention

**Fig. 2:**
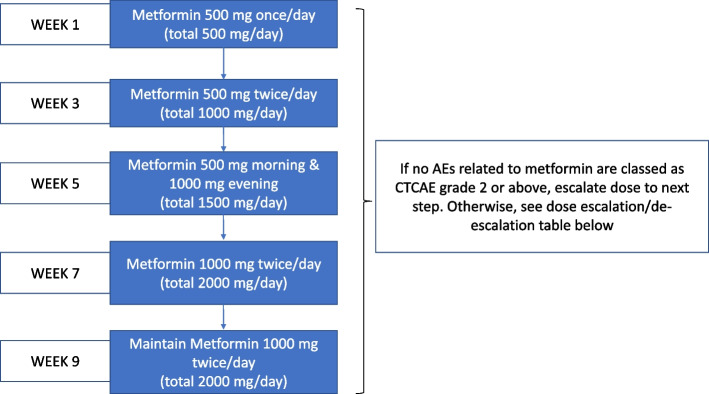
Dose escalation plan

Metformin immediate-release tablets, given initially as 500 mg once daily and dose increased every 14 days by 500 mg increments to 2g/daily (1000 mg b.d.) given orally (see Fig. 2) and continuously for up to 5 years. Dose titration will be based on having no adverse events related to metformin as classed as CTCAE Grade 2 or above.

#### Modifications

Dosing changes will be determined by adverse reactions measured by CTCAE grade (see Table 1).

**Table 1:** Dosing by adverse reaction (AR) CTCAE criteria

CTCAE grade (G)	Metformin dosing decision	2-week review
≤G1	Increase dose as per dose-titration plan, review in 2 weeks	Increase dose as per dose-titration plan
G2	Dosing decision to be made following review by Principal Investigator	If resolved to ≤G1, increase the dose as per the dose titration plan. If G2 ongoing, continue metformin at the current level and do not follow the titration plan
G3	Temporarily stop metformin Review in 2 weeks	If resolved to ≤G1 /G2, restart the metformin titration plan at lower dose of metformin. If on the lowest dose (500 mg od) metformin when experiencing toxicity, stop metformin permanently
G4	Discontinue metformin and repeat symptom check/institute medical management as appropriate	

#### Adverse events/serious adverse events

Adverse event (AE) monitoring starts from the consent until 30 days after the last dose of metformin (for participants in the metformin arm) and, for those in the control arm, their last trial visit. AEs will be graded according to CTCAE V5.0. All AEs will be reported via the clinical trial database to the OCTO Pharmacovigilance office. From consent up to the initial telemed call, AEs that are determined to be CTCAE grade 2 or above will be recorded in the Adverse Event CRF for all participants. At the initial telemed call and thereafter, ≥ grade 2 AEs will only be recorded in the adverse event CRF during the metformin titration phase. Grade 1 AEs are not required to be recorded as the safety profile of metformin is well established.

Serious AEs (SAE) will be collected from all participants from consent until the initial telemed call (post-randomisation). Any SAE that occurs in participants randomised to the control arm will not be defined as reportable and will be recorded in the clinical trial database. SAEs occurring in participants randomised to the metformin arm will be reported via the clinical trial database. Any suspected unexpected serious adverse reactions (SUSARs) will be reported to the Competent Authority and the Ethics committee by the OCTO Pharmacovigilance office.

#### Adherence

Participants will be asked to complete the MARS-5 questionnaire to assess metformin adherence [33]. If adherence is below the equivalent of 6 months over 5 years (10%) the participant will be non-evaluable. During the titration phase, metformin tolerance will be checked every 2 weeks via a telemed call by the research team based at the Oxford investigator (recruiting) site. Thereafter, adherence will be checked every 6 months via a telemed call using the MARS-5 questionnaire for the duration of the trial. Concomitant medication may be given as medically indicated.

#### Participant timeline

**Fig. 3:**
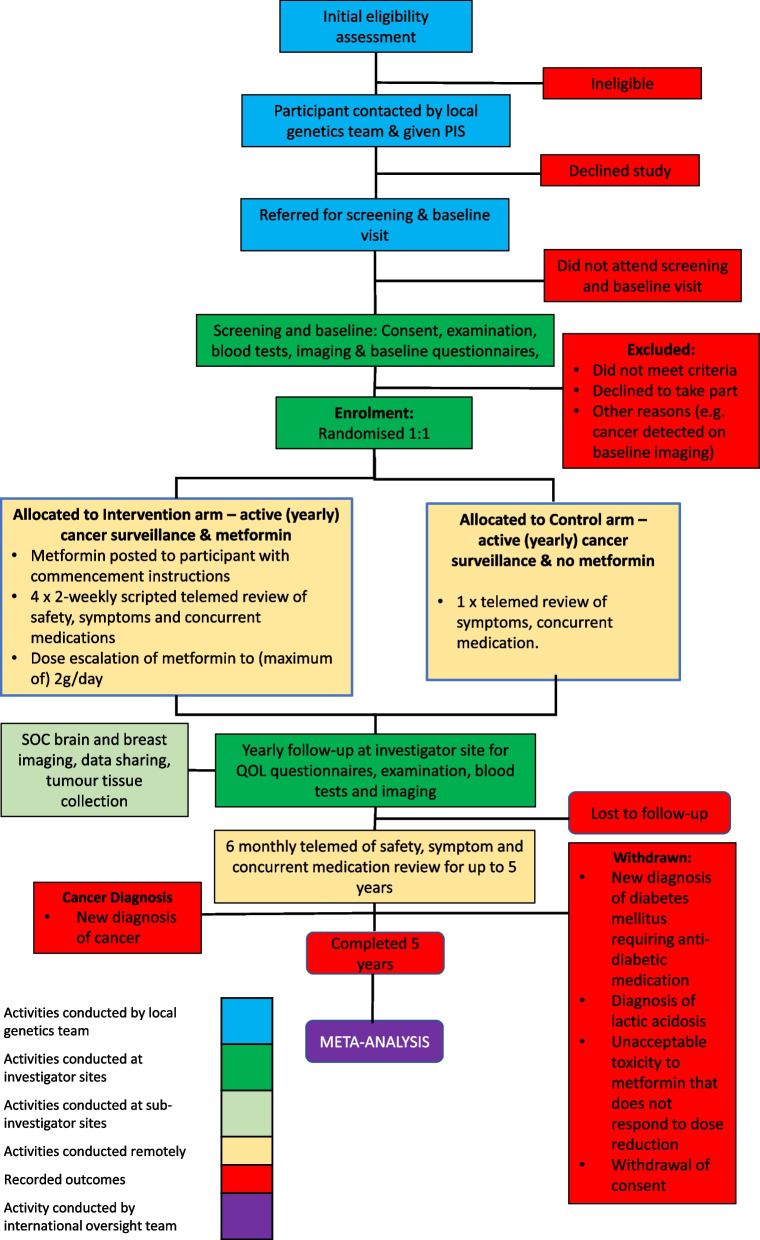
Participant flow diagram

The participant timeline is outlined in Fig. 3.

#### Outcomes: trial end-points

##### Primary end-point

Cumulative cancer-free survival up to 5 years (60 months) from randomisation between participants in the intervention (metformin) versus control (no metformin) arms. “Cancer” event defined as pathologically confirmed diagnosis of malignant cancer identified during trial participation or death from any cause.

##### Secondary end-points

i. Comparison of cumulative tumour-free survival at 5 years between trial arms. “Tumour” event including pathologically confirmed diagnosis of malignant cancer or clinically/scan detected benign or premalignant lesion death from any cause.
ii. Comparison of overall survival at 5 years between trial arms
iii. Comparison of clinical characteristics of emerging cancers between trial arms.
iv. Safety and toxicity of metformin
v. Acceptability of metformin
vi. Impact of metformin on quality of life (QOL)
vii. Impact of baseline lifestyle risk factors (e.g. weight, BMI, smoking, etc.) on cancer incidence

##### Exploratory end-points

1. To establish the mechanism of action of metformin as a cancer-preventative
  i. Whether baseline insulin sensitivity (using HOMA-IR as a surrogate marker) predicts cancer-free survival
  ii. Proportion of metformin’s chemoprevention effects that are indirect via insulin sensitivity (using HOMA-IR as a surrogate marker)
  iii. Proportion of metformin’s chemoprevention effects that are indirect via changes in circulating PI3K/AKT/mTOR signalling (using change to PI3K score).
  iv. Proportion of metformin’s chemoprevention effects that are direct via alterations to oxidative phosphorylation using OXPHOS gene signature as a surrogate marker.
2. To identify biomarkers of response or carcinogenesis
  i. Surrogate genetic and epigenetic markers of cancer-free survival or metformin response using next-generation sequencing of PBMCs and cfDNA
  ii. Proteomic markers of cancer-free survival or metformin response using reverse phase protein array of PBMCs.
  iii. Penetrance of *TP53* GPV in tumour tissue, correlating GPV with cancer diagnosis (location, stage and grade).
  iv. Retrospective identification or validation of circulating cancer biomarkers using mass spectroscopy/ELISA of serial plasma samples
3. To assess WB-MRI performance as a diagnostic tool for detecting cancers in participants with LFS by assessing yield and diagnostic accuracy of WB-MRI.

#### Sample size

Cancer incidence is affected by type and penetrance of *TP53* mutation [34] and is significantly reduced by prophylactic mastectomy. The SIGNIFY study [5] identified 13% of cancers after baseline imaging in 44 patients but, as many did not have preceding annual surveillance, this carries an inherent prevalence bias. As patients with cancers detected at baseline will be excluded from MILI, we referenced data collected between 2013 and 2017 from 1000 LFS adults by the international Li-Fraumeni Exploration (LiFE) Research Consortium [35]. These patients had undergone annual surveillance scans. This revealed an average yearly cancer incidence of 5% (unpublished). Therefore, we assume a 5% per year cancer incidence in our control arm.

The addition of metformin is estimated to reduce new cancer events by 50% at 5 years corresponding with a reduction in all-cause mortality by 20%. This assumes that 77% of participants will be cancer-free at 5 years in the control arm, and if the treatment reduces the incidence of cancer by 50%, 88.5% in the treatment arm will be cancer-free at 5 years. Were we to select a small effect size of, for example, 25%, we would need to recruit 1044 participants which would be unachievable given the LFS population and provide a median survival advantage of only 5 years in the metformin arm, equivalent to the duration of the study. Therefore we chose a higher effect size so data from this study could be used for the international meta-analysis powered to detect a lower risk reduction. This strategy was recently exemplified with pooling of data from the COVID-19 UK’s Recovery and WHO’s Solidarity trials to show the impact of hydroxychloroquine on survival [36]. The predicted median survival will be 28 years (from recruitment) in the metformin arm versus 13 years in the control arm. To detect this effect size with 80% power and one-sided log rank test with 5% error, 44 events should be observed. A sample size of 224 participants (112 in each arm) recruited over 2 years with a further 5 years of follow-up is therefore needed in order to observe this number of events with these assumptions. A 1% loss to follow-up rate and no treatment crossovers are also assumptions made.

### Assignment of interventions

#### Allocation

Participants are randomised 1:1 to one of two groups: surveillance plus metformin or surveillance alone. The control arm of surveillance alone without metformin (rather than surveillance plus placebo) was decided following a consultation and survey carried amongst the LFS community. Randomisation will be undertaken using minimisation, with minimisation factors: sex, age (16–25/26–35/36–45/46+ years), prior cancer, prior bilateral mastectomy and de novo versus familial mutTP53. The minimisation algorithm will be seeded with the initial participants using simple randomisation. A probabilistic element is included so minimisation is non-deterministic.

#### Implementation

Investigator (recruiting) site staff will confirm the diagnosis and the participant’s eligibility by completing the eligibility checklist within the trial randomisation form and randomising them to one of the two arms in the REDCap clinical trial database.

### Data collection, management and analyses

#### Data collection plan

In addition to baseline imaging and screening tests, all MILI participants undergo annual evaluation of fasting glucose and insulin levels to determine their insulin sensitivity (using the HOMA-IR score). Peripheral blood mononuclear cells (PBMCs) will be collected for next-generation sequencing of metabolic genes alongside proteomic measurement of PI3K/mTOR activity using reserve phase protein array (RPPA). Longitudinal plasma and cell-free DNA samples will also be collected so that carcinogenesis biomarkers can be retrospectively identified in those developing cancers during the study. In addition, tumour tissue will be sequenced to characterise *TP53* mutations and other relevant genetic changes. See Appendix Table 2 for full summary schedule of events.

Consent includes permission to conduct ancillary research on samples collected during the study. Researchers wishing to conduct appropriate and relevant research on the samples obtained during the study can request permission to access them via the Sample Access Committee which will be convened during the study.

#### Retention and follow-up post-withdrawal

For those who have permanently withdrawn from active trial participation early (not due to a cancer diagnosis), the investigator site will follow up on the participant’s health status with the local clinical team annually where possible for up to 5 years or until the trial ends (whichever comes first).

#### Data management

The full data management plan is available on request.

#### Statistical analysis

A detailed statistical analysis plan (SAP) has been drafted prior to the trial opening and will be finalised in advance of any primary outcome analysis. This plan will be reviewed by and receive input from the trial team and external committees (e.g. Data Safety Monitoring Committee) if appropriate.

All analyses will be on an intention-to-treat basis. Initial data analysis will be performed without knowledge of the randomisation status of participants. This means that participants will be analysed as they are randomised irrespective of the treatment actually received. Further sensitivity analysis populations will be defined in the SAP. Numerical and graphical summaries of all data will be presented including descriptions of missing data at each level. Kaplan-Meier curves will be produced by the intervention group for time-to-event outcomes.

Estimates of treatment effects will be reported with 95% CIs. The primary outcome will be analysed using a Cox proportional hazards regression model, with time-to-cancer event as the outcome; allocated group will be included as a covariate, as well as the minimisation factors (sex, age in years, prior cancer, prior bilateral mastectomy, de novo versus familial mutTP53). The proportional hazards assumption will be assessed graphically.

Details of secondary outcome analysis methods will be fully defined in the SAP but time-to-event outcomes will be analysed using the same principles as defined for the primary outcome.

A subgroup analysis of participants with de novo versus familial *TP53* GPV will be conducted as part of the MILI trial analysis. Pooled international analyses will be conducted after the completion of MILI as part of a separate trial.

Missing data will be minimised by careful data management. Missing data will be described with reasons given where available. The number and percentage of individuals in the missing category will be presented by treatment arm. All data collected in the electronic data capture system will be used, since only essential data items will be collected. No data will be considered spurious in the analysis since all data will be checked and cleaned before analysis. Analysis of exploratory end points may be performed separately from the final analysis as performed by the trial statisticians in accordance with the SAP.

### Monitoring

#### Data monitoring

An independent Data and Safety Monitoring committee will be formed for this trial. This committee will assess the trial data at least on an annual basis. The DSMC will make confidential recommendations to the Independent Trial Steering Committee. The Trial Steering Committee will be able to decide on stopping or continuing the trial or modifying the protocol.

#### Auditing

The trial will be subject to internal and external quality assurance audits to ensure compliance with the protocol and Good Clinical Practice requirements.

## Ethics and dissemination

MILI has been reviewed by West of Scotland Research Ethics Committee (REC) 1 (REC reference: 23/WS/0051) with minor changes requested and is now pending combined review approval from REC and Medicines and Healthcare Products Regulatory Agency (MHRA) and the Health Research Authority (HRA). Protocol amendments will be communicated to the relevant parties and through the dedicated MILI website.

### Consent

Informed consent will be obtained by suitably qualified and experienced named investigators. Study consent comprises agreeing to participate in the clinical study, permission for medical records held by the local genetics team to be shared with the recruiting hospital and telemedicine team, permission to donate blood and tissue samples, and for them to be used in other relevant research, and to allow de-identified MRI images to be used for teaching purposes during and after the trial.

### Data management and confidentiality

The trial will comply with the General Data Protection Regulation (GDPR) and Data Protection Act 2018, which require data to be de-identified as soon as it is practical to do so. Personal data recorded on all documents will be regarded as confidential. For the purpose of running the trial, the trial office will collect the participant’s name and contact details. This personal data will be stored in an area of the database which is separate from the clinical trial database. Access to the participant’s contact details will be limited to members of the trial team who need to contact the participant during the trial. All documents will be stored securely and only accessible by trial staff and authorised personnel. Axillary care, post-trial care or harm caused by the trial will be covered by the National Health Service provision of care.

### Dissemination

The trial results of the study will be communicated to participants, to the LFS community via the George Pantziarka TP53 Trust and to the scientific community via publication and presentation at scientific meetings. The Investigators will be involved in all stages of dissemination and will have final editorial control. The trial plans will be published online on the dedicated MILI website.

## Discussion

The MILI study is an example of a Precision-Prevention trial whereby a targeted cancer preventive is evaluated in a high-cancer-risk population for its impact on cancer incidence alongside translational aims of understanding its mechanism of action and exploring tumorigenesis biology. Metformin, a repurposed antidiabetic medicine, was selected as the cancer preventive for the MILI study after preclinical studies showed anticancer effect in *Trp53*-mutant mice. This was followed by a pilot study showing tolerability and acceptability in the target population at a standard dose (2 g/day). Hence, the study hypothesis is that people who carry *TP53* GPV will selectively benefit from metformin’s chemopreventive activity, even though the underpinning molecular mechanism is incompletely characterised, and will be explored in the study. Longitudinal sample collection from the 224 participants with LFS enrol into the MILI study will enable us to track changes in circulating biomarkers, such as anti-p53 antibodies, cell-free DNA and methylation profiling, comparing expression of these markers in those diagnosed with cancer versus those who remain cancer-free during the 5-year study. Sequencing of any arising tumours will provide important insights into the molecular pathogenesis of cancers occurring in *TP53* carriers as well as pathways of resistance to metformin.

The merger of datasets from other international centres running the MILI study (such as NIH which is adopting an identical protocol) will increase the statistical power by which we can explore *TP53* mutation phenotypes, i.e. whether certain *TP53* mutations correspond to specific cancer pathologies.

Most importantly, for individuals with LFS who have cancer risks significantly higher than the general population, this study is the first to explore a cancer preventive in this devastating condition. The rarity of LFS has hampered large randomised trials in the past but the Precision-Prevention design with its strong biological emphasis means that  fewer patients need to be enrolled. During study development, when the LFS charity, The George Pantziarka TP53 Trust, was polled to decide if an open-label or placebo-controlled study design was preferred, the former was chosen. If metformin proves effective, the evidence will be used to apply for metformin to be a NICE-approved chemopreventive for LFS. Currently, physicians can only prescribe the drug for type 2 diabetes and polycystic ovarian syndrome, even though there is considerable data linking it to cancer prevention.

The partnership with the LFS community in developing the protocol, national recruitment throughout the UK, the international data pooling and the ambition to embed translational research through it make MILI a flagship study in the field of innovative cancer prevention research. Not only will it provide important insights into a condition that has confounded understanding for many decades, but it will also provide an understanding of germ-line mutated *TP53*-driven tumorigenesis that will have implications for sporadic *TP53* mutated cancers. If positive, the use of a drug to reduce the inherent cancer risk associated with LFS will make a significant difference to the lives of people with LFS—who currently live with high levels of psychological stress and fear.

### Trial status

The trial opened for recruitment in December 2023. Recruitment is expected to be completed in December 2025.

Trial website: https://www.oncology.ox.ac.uk/clinical-trials/oncology-clinical-trials-office-octo/mili

Protocol Version 3.0, dated 13/09/23

*Trial Co-ordinating centre:*

Oncology Clinical Trials Office (OCTO)

Department of Oncology, University of Oxford

Old Road Campus Research Building

Roosevelt Drive, Headington

Oxford, OX3 7DQ

*Chief Investigator:*

Professor Sarah Blagden

*Principal Investigators:*

Dr Lara Hawkes

Prof Marc Tischkowitz

Dr Angela George

Dr Emma Woodward

Dr Louise Izatt

Dr Rachel Harrison

*Translational leads:*

Dr Simon Lord

Prof Xin Lu

*Radiology Lead:*

Dr James Franklin

*Statisticians*

Alexander Ooms

Maggie Qiao

*PPI Representatives:*

Dr Pan Pantziarka

Elizabeth Sam

Verity Easton

*Trial Management Group:*

Prof Sarah Blagden

Prof Gareth Evans

Dr Helen Hanson

Dr Lara Hawkes

Prof Marc Tischkowitz

Dr Angela George

Dr Emma Woodward

Dr Louise Izatt

Dr Rachel Harrison

Dr James Franklin

Alexander Ooms

Maggie Qiao

Dr Pan Pantziarka

Linda Collins

Rachel Shaw

Kendra Perez-Smith

*Data Monitoring and Safety Committee:*

Prof Shibani Nicum

Mr Nicholas Counsell

Prof Stuart Taylor

### Supplementary Information

**Additional file 1. **SPIRIT_Fillable-checklist.

## Figures and Tables

**Table 2:**
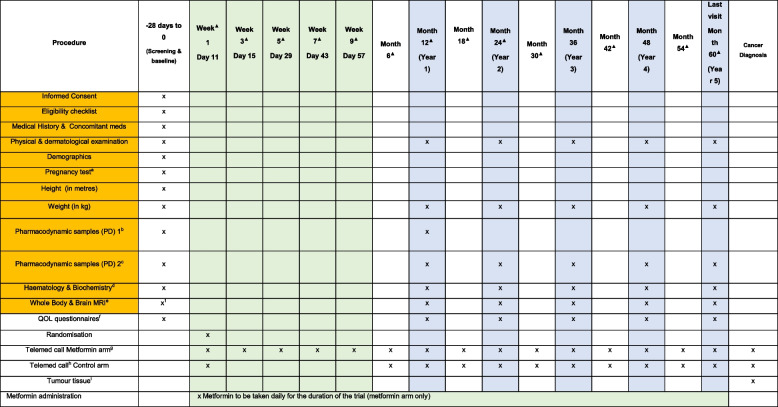
Summary of events

^a^Pregnancy test (urine) for participants of child-bearing potential and who are sexually active

^b^Pharmacodynamic sample PD1

^c^Pharmacodynamic sample PD2

^d^Fasting biochemistry and haematology blood: FBC (Hb, WBC, platelets), biochem (sodium, potassium, urea, creatinine, eGFR, liver function tests, vitamin B12 (baseline and year 5 only)) and for the translational research endpoints: insulin, glucose and IGF1 (baseline and M12)

^e^Whole body and brain MRI (WB-MRI) to be performed at the investigator (recruiting) sites. If a whole body and brain MRI scan has been completed in the 3 months prior to randomisation, this will be accepted as the baseline and screening scan.

^f^Quality of Life questionnaires: 12-item short form survey (SF12V2), Cancer Worry Scale & Treatment Burden Questionnaire (TBQ)

^g^Telemed call metformin arm*: Initial screening—explain randomisation outcome and call structure. Management of dose-titration phase, determine adverse events, changes to concomitant meds and review of adherence (MARS-5 Medication Adherence Questionnaire from month 6 onwards).

^h^Telemed call control arm*: Screening—explain randomisation outcome and call structure. From month 6 to determine adverse events, changes to concomitant meds.

^i^Tumour tissue: Paraffin-embedded diagnostic tumour tissue or pre-cancerous tissue block

^j^Metformin administration: metformin will be sent by post from the recruiting hospital pharmacy

*For both arms, additional telemed calls may be made by the telemed team

## Appendix

The participant information materials, informed consent form and laboratory manual are available on request from the corresponding authors.

^▲^Visit window

**Table:**

Call or visit	Window
Telemed call week 1	Within 3 working days of randomisation
Telemed call (dose titration phase)	Metformin start date + week 3/5/7/9 + within 3 working days
Telemed call months 6-60	+/- 1 week
Annual visit to investigator site	+/- 4 weeks
